# Highly Conserved *C*-Terminal Region of Indian Hedgehog *N*-Fragment Contributes to Its Auto-Processing and Multimer Formation

**DOI:** 10.3390/biom11060792

**Published:** 2021-05-25

**Authors:** Xiaoqing Wang, Hao Liu, Yanfang Liu, Gefei Han, Yushu Wang, Haifeng Chen, Lin He, Gang Ma

**Affiliations:** 1 Bio-X-Renji Hospital Research Center, Renji Hospital, School of Medicine, Shanghai Jiao Tong University, Shanghai 200240, China; wangxq0922@126.com; 2 Key Laboratory for the Genetics of Developmental and Neuropsychiatric Disorders (Ministry of Education), Bio-X Institutes, Shanghai Jiao Tong University, Shanghai 200240, China; liuyanfang287@163.com (Y.L.); gefee1@163.com (G.H.); violawys@sjtu.edu.cn (Y.W.); 3 State Key Laboratory of Microbial Metabolism, Department of Bioinformatics and Biostatistics, National Experimental Teaching Center for Life Sciences and Biotechnology, School of Life Sciences and Biotechnology, Shanghai Jiao Tong University, Shanghai 200240, China; chaohao2010@sjtu.edu.cn

**Keywords:** Indian Hedgehog, *C*-terminal region, autoproteolytic cleavage, protein stability, multimer formation

## Abstract

Hedgehog (HH) is a highly conserved secretory signalling protein family mainly involved in embryonic development, homeostasis, and tumorigenesis. HH is generally synthesised as a precursor, which subsequently undergoes autoproteolytic cleavage to generate an amino-terminal fragment (HH-N), mediating signalling, and a carboxyl-terminal fragment (HH-C), catalysing the auto-processing reaction. The *N*-terminal region of HH-N is required for HH multimer formation to promote signal transduction, whilst the functions of the *C*-terminal region of HH-N remain ambiguous. This study focused on Indian Hedgehog (IHH), a member of the HH family, to explore the functions of the *C*-terminal region of the amino-terminal fragment of IHH (IHH-N) via protein truncation, cell-based assays, and 3D structure prediction. The results revealed that three amino acids, including S195, A196, and A197, were crucial for the multimer formation by inserting the mutual binding of IHH-N proteins. K191, S192, E193, and H194 had an extremely remarkable effect on IHH self-cleavage. In addition, A198, K199, and T200 evidently affected the stability of IHH-N. This work suggested that the *C*-terminus of IHH-N played an important role in the physiological function of IHH at multiple levels, thus deepening the understanding of HH biochemical properties.

## 1. Introduction

The Hedgehog (HH) family is well known for its essential role in the normal embryonic development of invertebrates and vertebrates and the maintenance of adult tissue homeostasis [1,2]. Deregulation of its signalling pathway leads to the destruction of tissue homeostasis and stem cell self-renewal, further resulting in the occurrence of cancer [3,4]. In higher vertebrates, the HH family mainly contains three homologous proteins: Sonic Hedgehog (SHH), Indian Hedgehog (IHH), and Desert Hedgehog (DHH) [5]. Although their expression patterns and physiological functions are different, their protein processing and signal transduction exhibit high similarities.

HH is regularly synthesised as a precursor in the endoplasmic reticulum of the producing cells, and it undergoes autoproteolysis to generate a functional HH-N, mediating signalling, and a HH-C, catalysing the auto-processing reaction [6,7]. A cholesterol molecule attacks the self-cleavage site to promote the self-cleavage reaction, and binds to the *C*-terminus of HH-N after cleavage. Subsequently, palmitic acid attaches to the *N*-terminal of HH-N to produce a mature signalling ligand, HHNp [8]. With the help of Dispatched, a transmembrane protein, and heparan sulphate proteoglycans (HSPGs), HHNp is released to the extracellular matrix and forms a soluble multimeric complex, s-HHNp, to facilitate movement. After reaching the recipient cells, s-HHNp binds with the receptor Patched (PTC), relieving the inhibition towards Smoothened (SMO) and further activating the downstream transcription factors GLIs, which trigger the expression of target genes, such as *Ptch1* and *Gli1* [9].

The *N*-terminus of HH-N is highly conserved in homologous proteins and various species [10]. Moreover, the palmitic acid modification site [8], HSPG binding sites [11], and PTC binding sites [12] are located at the *N*-terminus. Three regions, including amino acids 27–34, amino acids 35–48, and the palmitate acceptor site C25 in the *N*-terminus of SHH-N, are necessary for multimer formation [13]. The previous study depicted that G31R in the *N*-terminus of SHH-N affects protein stability [14]. These results suggest that the conserved *N*-terminus of HH-N plays an important role in signal transduction.

However, the function of the *C*-terminus of HH-N is poorly studied. By re-analysing the previously reported IHH crystal structure [15], a 3D structural model of the self-interaction of IHH was discovered. It showed that the S195, A196, and A197 located in the *C*-terminus of IHH-N could bind to the interior of another IHH molecule. Interestingly, a similar intermolecular interaction model was also found in SHH [16], although the function of this intermolecular interaction remains unclear. In addition, by aligning the amino acid sequences of IHH in different species, the homology of S195, A196, and A197 and the adjacent amino acids were almost 100% (Figure S1), which also indicates the potential conserved biological functions of such regions.

In this research, the conserved region of the IHH-N *C*-terminus was studied by analysing different truncations and point mutations to elucidate the functions of the *C*-terminus. The results showed that three amino acids, namely, S195, A196, and A197, were crucial for IHH-N multimerization. Meanwhile, K191, S192, E193, and H194 had an extremely substantial effect on IHH self-cleavage. A198, K199, and T200 evidently affected the stability of IHH-N. Models of IHH-N intermolecular interaction and the potential mechanism of full-length IHH self-cleavage were also proposed on the basis of 3D structure, which is important for deepening the understanding of IHH biochemical properties.

## 2. Materials and Methods

### 2.1. Construction of HH Mutants

Human *IHH* cDNA was cloned and inserted into the pIND (Sp1)-based inducible expression vector (Invitrogen, Carlsbad, CA, USA) with double FLAG-tags were introduced into the IHH-N as described previously [15], or the pCMV-based expression vector (Promega, Madison, WI, USA) with double FLAG-tags were introduced into the IHH-C (pCMV-C-2flags). The mutations were introduced through PCR, employing truncation or single-nucleotide substitution primers. The cDNA of human *SHH* was amplified from the pLNB-SHH plasmid which was provided by Dr. JM Mason from the Gene Therapy Vector Laboratory (Manhasset, NY, USA). Then, the *SHH* cDNA was mutated and inserted into the pcDNA3.0-6xMyc vector. In fusion expression protein constructs, the cDNA of enhanced green fluorescent protein (*EGFP*) was cloned from the pEGFP-C3 vector and inserted into the pCMV-C-2flags vector with IHH-C. All constructs were confirmed by Sanger sequencing. The primers used in this study are listed in Table S1.

### 2.2. Construction of Stable Cell Line and Gel-Filtration Chromatography

The WT or SAA truncated (ΔSAA) plasmid was transiently transfected into the EcR-CHO (ECHO) cells (Invitrogen, Carlsbad, CA, USA) using Lipofectamine 3000 (Invitrogen). Twenty-four hours (24 h) later, the cells were seeded in a six-well plate via limiting dilution method and cultured in the screening medium (G418, 800 ng/μL, Sigma-Aldrich, St. Louis, MO, USA). After 2–3 weeks, the monoclones were picked for cultivation and detection of IHH-N expression, then the best clone was selected to expand the culture. After filling in a single-cell layer, ponasterone A (5 μg/mL, Santa Cruz Biotechnology, Santa Cruz, CA, USA) was added for another 24 h cultivation. The supernatants were collected and concentrated immediately with Amicon Centricon-4 centrifugal filter devices (Millipore, Bedford, MA, USA) before addition of protease inhibitors (Yeasen, Shanghai, China). After automatic chromatography (Superdex 200 column, GE Company, Boston, MA, USA), the collected aliquots were concentrated by trichloroacetic acid and immunoblotted with anti-FLAG antibody (Invitrogen, MA1-91878, 1:1000; Sigma-Aldrich, F3165, 1:4000) and goat anti-mouse secondary antibody (ThermoFisher Scientific, Waltham, MA, USA, 31430, 1:5000).

### 2.3. IHH Activity Assay in C3H10T1/2 Cells

The C3H10T1/2 cells (ATCCs) were seeded in twenty-four-well plates with an appropriate density and cultured in a growth medium containing DMEM, 10% foetal bovine serum, and 1% penicillin/streptomycin. The culture supernatants of WT or ΔSAA stable cell line were added into the growth medium and continued to incubate at 37 °C for 4–5 days. Subsequently, the C3H10T1/2 cells were washed in cold PBS and the IHH-N activity was measured using an alkaline phosphatase (AKP) substrate kit (Vector labs, Burlingame, CA, USA, SK-5100). All induction assays were performed in triplicate.

### 2.4. Transfection and Analysis of HH Mutants

pIND-based plasmids were transfected into the ECHO cells by using Lipofectamine 3000 to study the expression of IHH protein. After 24 h, ponasterone A was added to the cell culture medium. Another 24 h later, the cells were lysed using lysis buffer (Beyotime Shanghai, China) with addition of a protease inhibitor. The cell homogenates were cleared by centrifugation, then separated by 12% SDS-PAGE and immunoblotted with anti-FLAG monoclonal antibody. Similarly, the pCMV-based IHH constructs were transiently transfected into ECHO cells and 48 h later, the cells were lysed and detected with the same antibodies. The pcDNA3.0-6xMyc plasmids were transfected into the HEK293T (293T) cells (ATCCs) and detected using anti-SHH (Santa Cruz Biotechnology, sc-365112, 1:500) and anti-Myc (Invitrogen, 13–2500, 1:500) monoclonal antibodies. The fusion expression plasmids were transfected into the 293T cells, immunoblotted with anti-FLAG monoclonal antibody and anti-EGFP polyclonal antibody (Abcam, Cambridge, MA, USA, ab5450, 1:5000). Anti-GAPDH HRP-conjugated antibody (Proteintech, Rosemont, IL, USA, HRP-60004, 1:5000) was chosen as the internal control for Western blot. The secondary antibodies used in this experiment were goat anti-mouse IgG (H+L) and rabbit anti goat IgG (H+L) (Invitrogen, A27014, 1:2000). The grey value of the protein bands was analysed by Image J. The ratio of IHH-N (or IHH-C) to the total IHH protein, which is composed of full-length protein and IHH-N (or IHH-C), was calculated with the WT as the standard. Statistical analysis of relative expression was calculated by SPSS.

### 2.5. Prediction of IHH Protein Structure

The software I-TASSER server with default parameters was used for all structural predictions [17]. The predicted structures of the mutated IHH-N and SHH-N were based on the existing structure (PDB ID: 3K7G and 3N1R). The predicted structures of IHH-C and SHH-C were aligned to the crystal structure of HH-C protein from *Drosophila melanogaster* (PDB ID: 1AT0) by using PyMOL software. The raw and optimised Ca root-mean-square deviations (RMSDs) were calculated by PyMOL. The structures of EGFP-IHH fusion proteins with different mutations were predicted on the basis of the structure of the existing EGFP (PDB ID: 2Y0G) and predicted IHH-C.

### 2.6. Cell Immunofluorescence of IHH Mutants

The WT, ΔSAA, KSEH truncation (ΔKSEH), and cholesterol modification site mutation C203* plasmids were transfected into ECHO cells as described above. Next, 24 h after ponasterone A was added, anti-FLAG monoclonal antibody was incubated at 37 °C for 2 h to stain the IHHNp on the cell membrane surface. Then, cells were fixed with 80% cold acetone for 30 min at 4 °C and labelled with goat anti-mouse Alex488 (Invitrogen, A32723, 1:1000) for 2 h at 37 °C. A Leica SP8 laser scanning microscope was used for detecting immunofluorescence.

## 3. Results

### 3.1. S195, A196, and A197 Are Involved in Multimerization of IHH-N Protein by Maintaining Intermolecular Interactions

**Figure 1:**
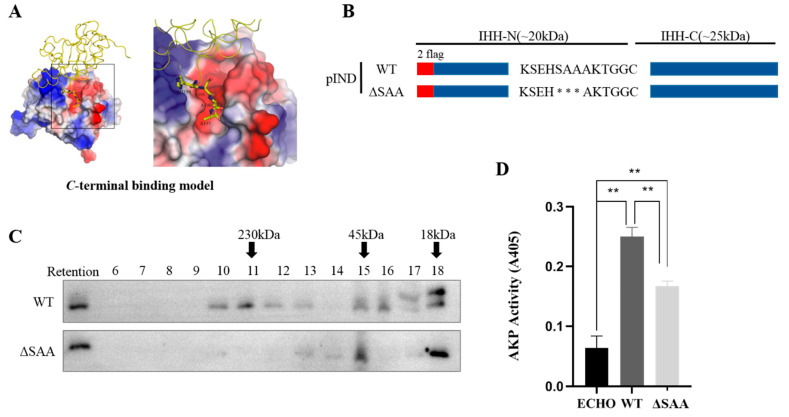
S195, A196, and A197 are required for multimer formation of IHH-N. (**A**) The *C*-terminal binding model of IHH-N showed that the S195, A196, and A197 of molecule A (pictured as backbone trace) were positioned at the zinc binding site of molecule B (pictured with the molecular surface area coloured by electrostatic potential). (**B**) Schematic of WT and SAA truncation. * truncated amino acids. (**C**) The multimeric form of IHH-N was analysed using gel-filtration chromatography. The retention was the total protein collected in the cell culture supernatants. The amount of multimers in fractions 10–13 in ΔSAA were significantly reduced compared with that in the WT. (**D**) The results of C3H10T1/2 cell differentiation showed that the AKP activity was significantly impaired in ΔSAA, suggesting a remarkable attenuation of the IHH signalling activity. Data are mean ± s.e.m, *n* = 3, ** *p* < 0.01.

In the previous research, the crystal structures of human WT and three brachydactyly type A1 (BDA1)-related mutant IHH-N protein domains were resolved to further understand the biochemical consequences of these mutations [15]. Re-analysis of the WT IHH-N crystal structure revealed two interaction models between two symmetry-related molecules of IHH-N: one was the *C*-terminal binding model where the S195, A196, and A197 located in the *C*-terminus of molecule A were positioned at the zinc binding site of molecule B (Figure 1A), and the other was the *N*-terminal binding model, where the amino terminus (R39, P40, P41, R42, and K43) of molecule A was located at the zinc binding site of molecule B (Figure S2). These two models have been reported in SHH-N [13,16] and the *N*-terminal interaction model was considered to be required for multimeric complex formation [13].

However, whether *C*-terminus, especially S195, A196, and A197, is vital for the multimer formation of IHH-N remains unclear. Gel filtration chromatography showed that the ΔSAA had different elution profiles compared with the WT (Figure 1B,C), especially a substantially reduced multimer in fractions 10–13 in ΔSAA. However, no apparent change in monomer was found in fractions 17 and 18 (Figure 1C). This result indicated that S195, A196, and A197 were crucial for the multimer formation of IHH-N proteins.

Given that HH signalling activity is associated with the multimer level, the influence of ΔSAA on HH signalling activity was elucidated using cell differentiation assay in vitro. The C3H10T1/2 cell line is one of the mesenchymal stem cells that could be differentiated into diverse terminal cells under the stimulation of distinct biological molecules. After responding to HH ligands, these cells are differentiated into mature osteoblasts, and express the corresponding marker AKP. The culture supernatants of the WT or ΔSAA stable cell line were collected and added into the medium of C3H10T1/2 cells, then the expression of AKP was detected after 5 days. The results clearly showed that the AKP activity was significantly impaired in ΔSAA (Figure 1D), suggesting a significant attenuation of the IHH signalling activity. Therefore, all results indicated that S195, A196, and A197, which were located in the *C*-terminus of IHH-N, were essential for multimerization to facilitate signalling activity.

### 3.2. Conserved C-Terminus of IHH-N Fragment Affects Protein Self-Cleavage and Stability

The amino acid sequences of different species were aligned to further clarify the potential functions of the IHH-N *C*-terminus. The results showed that the homology of S195, A196, and A197 and adjacent amino acids were almost 100% (Figure S1A), indicating the potential conserved biological functions of such region.

**Figure 2:**
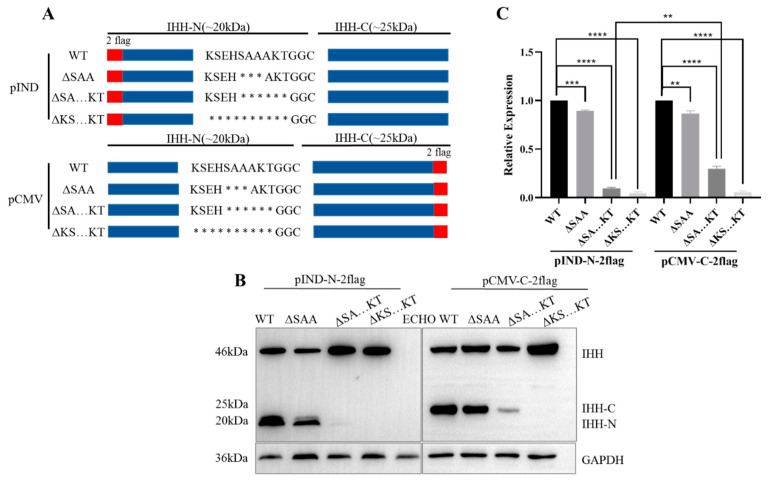
Conserved *C*-terminal region of IHH-N affects protein self-cleavage and stability. (**A**) Schematic of different truncations. * truncated amino acids. (**B**,**C**) Western blot and quantitative statistical analysis of the different truncations. The grey value of the protein bands was analysed by Image J. The ratio of IHH-N (or IHH-C) to the total IHH protein, which is composed of full-length protein and IHH-N (or IHH-C), was calculated with the WT as the standard. Data are mean ± s.e.m, *n* = 3, ** *p* < 0.01, *** *p* < 0.001, **** *p* < 0.0001. The IHH-N and IHH-C fragments were slightly reduced in ΔSAA and remarkably less in ΔSA…KT than that in WT. IHH-N and IHH-C were not detected completely in ΔKS...KT.

Ten amino acids (KSEHSAAAKT), including S195, A196, and A197, were initially selected to construct different truncations, and the relevant expression of IHH-N and IHH-C was studied (Figure 2A). The results showed that the IHH-N and IHH-C fragments were slightly reduced in ΔSAA but remarkably less in SAAAKT truncation (ΔSA…KT) than that in the WT. In addition, neither IHH-N nor IHH-C was identified in KSEHSAAAKT truncation (ΔKS…KT, Figure 2B,C).

Once self-cleavage occurs, the IHH-N and IHH-C fragments are generated simultaneously, which means that the occurrence of self-cleavage could be manifested by detecting either of these two fragments. When deleting S195, A196, and A197, the self-cleavage was only slightly affected and IHH-N proteins were still stable. The deletion of six amino acids (SAAAKT) exerted a modest effect on the self-cleavage according to the partial reduction in IHH-C protein. However, the expression of IHH-N was significantly reduced compared with that of IHH-C, indicating that the lack of A198, K199, and T200 may decrease protein stability and aggravate protein degradation. Moreover, when all 10 amino acids were deleted, neither IHH-N nor IHH-C could be detected, implying that amino acids K191, S192, E193, and H194 were involved in the autoproteolytic cleavage reaction of full-length IHH precursor. In total, the three regions had effects on protein self-cleavage or stability to varying degrees. Further research was conducted on these regions to confirm this effect in detail.

### 3.3. K191, S192, E193, and H194 Synergistically Affect Self-Cleavage of IHH Precursor

**Figure 3:**
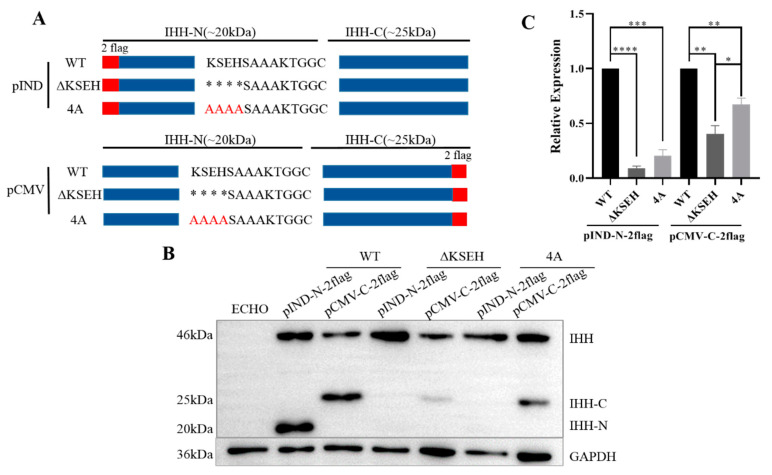
K191, S192, E193, and H194 are required for IHH self-cleavage. (**A**) Schematic of ΔKSEH and 4A constructs. Red letters indicate replaced amino acids; * truncated amino acids. (**B**,**C**) Western blot and statistical analysis showed that IHH-N was absent in ΔKSEH and 4A compared with WT, whereas IHH-C was remarkably reduced, indicating that IHH self-cleavage was considerably affected. Data are mean ± s.e.m, *n* = 3, * *p* < 0.05, ** *p* < 0.01, *** *p* < 0.001, **** *p* < 0.0001.

The results of ΔKS…KT revealed that K191, S192, E193, and H194 may play a decisive role in IHH precursor self-cleavage. IHH-N was expectedly absent and IHH-C was substantially reduced in the corresponding KSEH truncation (Figure 3A–C). Then, KSEH was replaced with AAAA (Figure 3A) to rule out the possibility that the destroyed self-cleavage could be attributed to the drastic change in the spatial structure after KSEH deletion. The result clearly proved that although the expression of IHH-C slightly increased, it was still far lower than that of WT, indicating that self-cleavage was also considerably inhibited. Thus, self-cleavage failure resulted from KSEH deficiency but not from severe disruption in protein structure.

**Figure 4:**
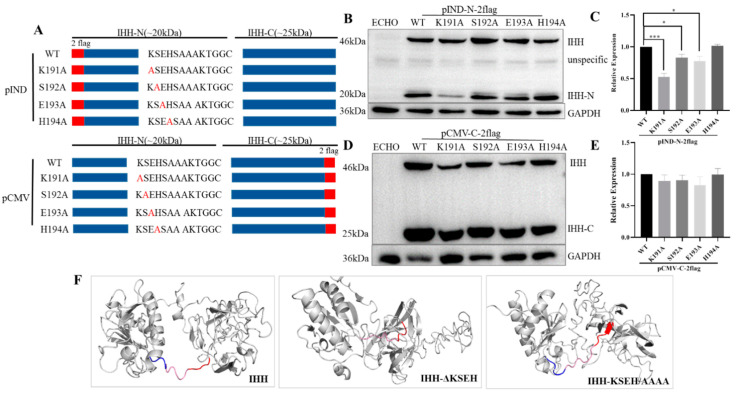
Analysis of point mutations in KSEH region and structural simulation of KSEH mutations. (**A**) Schematic of point mutations in KSEH. Red letters indicate mutant amino acid. (**B**,**C**) Western blot and statistical analysis showed that IHH-N could be detected in the corresponding point mutant constructs, but it was obviously reduced in K191A mutation and slightly reduced in S192A and E193A mutations, indicating that these amino acids may have a fractional effect on protein stability. Data are mean ± s.e.m, *n* = 3, * *p* < 0.05, *** *p* < 0.001. (**D**,**E**) Western blot and statistical analysis (*n* = 3) showed that the expression levels of IHH-C were statistically similar to those of WT, suggesting that KSEH worked together for IHH self-cleavage. (**F**) Structural simulation of IHH protein showed that the *C*-terminal region, including cholesterol modification site G202 (blue, KSEH; pink, SAAAKT; and red, GGCF), was presented on the surface as α-helices in WT IHH but covered and changed to an aperiodical coil in KSEH truncation and KSEH/AAAA replacement. This finding indicated that KSEH was critical for the surface presentation of the self-cleavage site on IHH protein.

Subsequently, four-point mutations were constructed to determine which amino acid plays a decisive role in K191, S192, E193, and H194 (Figure 4A). The results showed that IHH-N and IHH-C were present in these mutants (Figure 4B,D) and all expression levels of IHH-C were similar to those of the WT (Figure 4E), suggesting that KSEH worked together for IHH self-cleavage instead of being only dependent on a single amino acid. Unexpectedly, the IHH-N protein was obviously reduced in K191A mutation and slightly reduced in S192A and E193A mutations (Figure 4B,C), indicating that these three amino acids may not only be involved in autoproteolytic cleavage but also in protein stability maintenance. This conclusion further explained the phenotype of the complete disappearance of the IHH-N fragment but a weak expression of the IHH-C fragment in KSEH truncation and KSEH/AAAA replacement assays (Figure 3B).

The occurrence of self-cleavage typically depends on the attack of cholesterol on the thioester bond at the cleavage site [18]. Naturally, the cleavage site must be properly present on the protein surface to facilitate the attack (Figure 4F). The 3D structures of full-length IHH with ΔKSEH and KSEH/AAAA mutations were predicted using I-TASSER software, which was also used for the following SHH protein predictions, to determine whether KSEH affects the presentation of the self-cleavage site. The structures of IHH-N and SHH-N came from the existing structure (PDB ID: 3K7G and 3N1R). Meanwhile, the robustness of the predicted human IHH-C and SHH-C structures with the published structure of drosophila HH-C [19] (PDB 1AT0) were calculated using PyMOL to ensure the reliability of modelling. The raw Ca RMSDs for IHH-C and SHH-C were 1.549 and 2.585 Å, respectively, whilst the optimised Ca RMSDs were 0.453 and 0.823 Å, respectively, indicating that their structures were similar (Figure S3A).

As expected, the cleavage site, especially the cholesterol-modified amino acid G202, was trapped inside the spherical IHH-C in ΔKSEH and KSEH/AAAA mutations (Figure 4F). This finding indicated that the probability of cholesterol collision and self-cleavage were substantially reduced. Instead, the cleavage site was normally presented on the surface of the predicted structure of WT, ΔSAA, and AKT truncation (ΔAKT) (Figure S3B).

In order to further confirm whether KSEH is decisive for the presentation of the cholesterol modification site, IHH-N was directly replaced with EGFP and the highly conserved *C*-terminus was retained to construct different fusion proteins (Figure S4A). Contrary to the expectation, deleting K191, S192, E193, and H194 did not affect the self-cleavage of EGFP-IHH fusion precursor at all, even though the 10 conservative amino acids were deleted simultaneously (Figure S4B–D), and this finding was identical with that in a previous EGFP-SHH fusion protein study [20]. The predicted structures of fusion proteins indicated that IHH-N and EGFP presented relatively independent and intact spatial structures, with the self-cleavage site exposed on the surface all the time (Figure S4E), regardless of whether KSEH was truncated or even if 10 amino acids were knocked out. This result demonstrated that the normal presentation of the cholesterol modification site depended not only on the KSEH region but also on the whole spatial structure of the IHH protein.

### 3.4. K186, A187, E188, and N189 Also Severely Affect SHH Self-Cleavage by Obstructing the Presentation of the Cholesterol Modification Site

**Figure 5:**
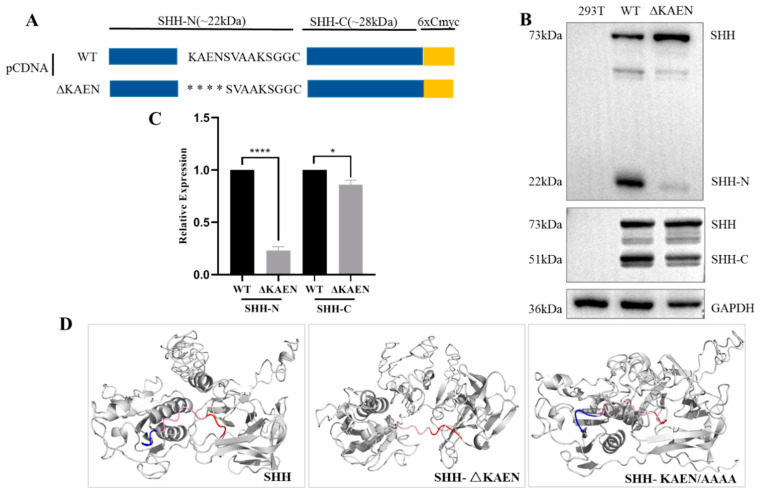
K186, A187, E188, and N189 affect SHH self-cleavage. (**A**) Schematic of WT and ΔKAEN constructs of SHH. * truncated amino acids. (**B**,**C**) Western blot and statistical analysis showed that SHH-N almost disappeared, and the expression of SHH-C decreased remarkably when KAEN was knocked out. Data are mean ± s.e.m, *n* = 3, * *p* < 0.05, **** *p* < 0.0001. (**D**) Structural simulation of SHH protein showed that the *C*-terminal region, including self-cleavage site between G200 and C201 (blue, KAEN; pink, SVAAKS; red, GGCF) was presented on the surface as α-helices in WT but covered and changed to an aperiodical coil in KAEN truncation and KAEN/AAAA replacement. This finding indicated that the KAEN region was critical for the surface presentation of the cholesterol modification site, similar to that of IHH.

SHH, another member of the HH family, also has a conserved SHH-N *C*-terminal sequence (Figure S1) and a highly conserved domain consisting of K186, A187, E188, and N189 corresponding to K191, S192, E193, and H194 at the same position in IHH. A KAEN truncation (ΔKAEN) was constructed (Figure 5A) to find out whether KAEN affected the self-cleavage as KSEH did. SHH-N almost disappeared, whilst SHH-C decreased to a certain degree, which was similar to that of IHH (Figure 5B,C). The structure of SHH was subsequently predicted, and the results revealed that deleting KAEN or replacing KAEN with AAAA could similarly disrupt the presentation of the self-cleavage site (Figure 5D). Overall, these results indicated that even though this region was not completely conserved in the HH family, it affected self-cleavage in an analogical manner.

### 3.5. A198, K199, and T200 Evidently Affect the Stability of IHH-N

**Figure 6:**
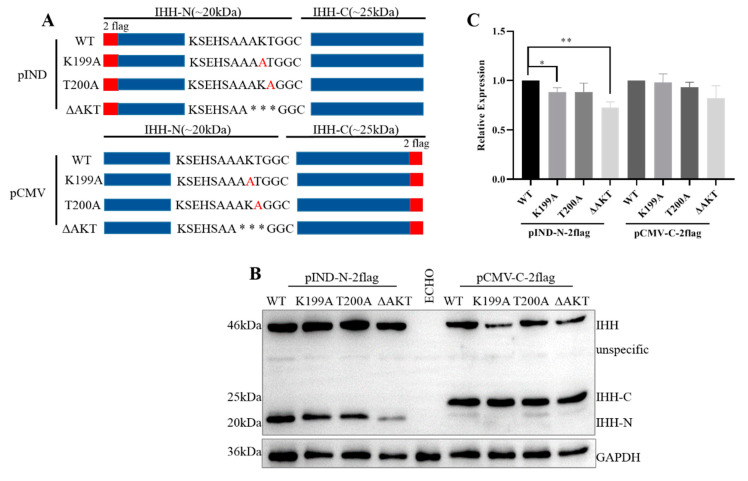
A198, K199, and T200 affect the stability of IHH-N. (**A**) Schematic of point mutations and truncations. Red letters indicate point-mutation amino acids; * truncated amino acids. (**B**,**C**) Western blot and statistical analysis showed that the expression of point mutations were roughly identical to those of WT. After A198, K199, and T200 were deleted, the normal expression of IHH-C revealed that the self-cleavage was not affected, whereas the decrease in IHH-N indicated that the stability may be affected. Data are mean ± s.e.m, *n* = 4, * *p* < 0.05, ** *p* < 0.01.

Point mutations and truncation were developed in the AKT region (Figure 6A), and the expression of IHH-N and IHH-C were characterized to further clarify the effect of A198, K199, and T200 on protein stability and self-cleavage. The point mutations K199A and T200A showed almost similar expression levels of IHH-N and IHH-C compared to that of WT (Figure 6B,C), indicating that mutating a single amino acid did not affect the stability of IHH-N. However, when three amino acids were deleted altogether, the expression of IHH-C was normal but that of IHH-N remarkably decreased, thereby revealing that A198, K199, and T200 acted as a combination to affect protein stability.

## 4. Discussion

IHH is one of the secreted ligands in the HH signalling pathway, and it plays an important role in regulating cartilage formation and development [5]. Missense mutations in *IHH* lead to skeletal developmental defects, such as BDA1 [21] and acrocapitofemoral dysplasia (ACFD) [22]. The *N*-terminus of HH-N plays a vital role in multimer formation, whilst the physiological functions of the *C*-terminus of HH-N have not been elucidated.

The structure of SHH-N was first reported in 1995 [16], and the *C*-terminus of one SHH-N (A194 and K195) was revealed to be positioned at the zinc-binding region of another SHH-N. This interaction might contribute to maintaining zinc binding and the potential hydrolytic activity of SHH-N [16], although the hydrolase activity is not required for Hh signalling transduction [23]. However, zinc is indeed essential for HH protein stability and signalling activity [23,24]. Moreover, zinc makes a vital contribution to the binding between HH and Hhip, which is a highly conserved and vertebrate-specific inhibitor of Hh signalling [25]. A recent study revealed that the cholesteryl moiety associated with seven amino acids (_191_VAAKSGG_197_) in the *C*-terminus of SHH-N is embedded in a cavity of receptor PTC1 to relieve the inhibition of SMO by PTC1 and stimulate the Hh signalling pathway [26]. All the above studies implied that the *C*-terminus of HH may exert multiple vital functions to regulate protein stability, partner binding, and signalling activity.

In this study, the highly conserved *C*-terminus of IHH-N was divided into three regions to analyse their functions in self-cleavage, protein stability and multimer formation. The functions of these three regions overlap one another but have their own emphasis. For example, K191, S192, E193, and H194 significantly affected the autoproteolytic cleavage reaction by obstructing the presentation of the cholesterol modification site. A198, K199, and T200 mainly affected the stability of IHH-N. In addition, inserting S195, A196, and A197 in another adjacent IHH-N molecule contributed to IHH-N multimer formation.

Under physiological conditions, the IHH protein is attacked by a cholesterol molecule to promote the self-cleavage reaction and form two different fragments. The relatively stable IHH-C was normal in the ΔAKT but slightly reduced in the ΔSAA. A more drastic decrease occurred in the ΔKSEH, indicating that the autoproteolytic cleavage reaction was obstructed to a great extent, which demonstrated that the KSEH region was the most critical region for self-cleavage. Similar discoveries were found in SHH. Subsequently, the possible mechanism was clarified through structural prediction and the results showed that the cholesterol modification site could not be normally presented on the protein surface in ΔKSEH, which then reduced the probability of cholesterol colliding with the self-cleavage site and caused the self-cleavage reaction unsuccessfully. However, it cannot be ruled out the possibility that the KSEH deletion or substitution changes the IHH structure, causing self-cleavage to be affected; thus, further study is needed.

However, the EGFP replacement assay illustrated that the KSEH region was not decisive to the self-cleavage, and the overall structure of the IHH-N had a considerable effect. In addition, the self-cleavage was slightly affected in ΔSAA and ΔSA…KT, possibly due to the mild changes of the polypeptide chain surrounding the cholesterol modification site. All results strongly indicate that the presentation of the self-cleavage site was dependent on the KSEH region and 3D structure of the whole IHH-N protein.

This mechanism expands the understanding of the factors that influence protein self-cleavage. Two types of factors affecting autoproteolytic cleavage have been proposed prior to this study. One factor is cleavage site mutations, such as C203A, which affect the formation of thioester bonds and prevent self-cleavage from occurring [27]. The other one is HH-C active centre mutations, such as H329Y, which destroy the activity of HH-C as a cholesterol transferase; thus, the HH protein could not be self-cleaved [6]. Another study has shown that mutations or deletions of certain amino acids in the *N*-terminus of SHH-N also affect the self-cleavage [13], but the reason remains unclear. This, we predicted that these amino acids may also be involved in maintaining the correct presentation of the self-cleavage site or the complete 3D structure of IHH-N.

In this research, it was found that ΔAKT did not affect the self-cleavage but significantly affected the protein stability. Unfortunately, the potential mechanism could not be predicted due to the lack of AKT in the existing SHH and IHH protein structures because of AKT hydrolysis in crystallisation [15,16]. In fact, a large number of studies have found that mutations in different regions of the HH-N would reduce protein stability. For example, G31R in the *N*-terminus of SHH-N [14], BDA1-related mutations in the middle of IHH-N [15], and ΔAKT in the *C*-terminus of IHH-N in this study. In addition, we also found that ACFD-associated mutation V190A [22], which is closed to the KSEH in the *C*-terminus of IHH-N, affected the self-cleavage slightly but reduced protein stability obviously (Figure S5), further supporting the significance of the IHH-N *C*-terminus and also implying that V190A mutation may cause disease in part by reducing the IHH-N protein level.

Several studies tried to clarify the potential mechanism of the decrease in protein stability. Traiffort et al. found that the eight mutations on SHH, located near the narrow gap bound to zinc, cause a significant decrease in SHH-N [14]. Three of the eight amino acids are located in the zinc-binding region, indicating that the mutations of these three amino acids affect zinc binding and reduce the stability of SHH-N. Although the other five amino acids are not in this region, reducing the stability by affecting zinc binding indirectly is possible. The other two studies found that E95K and D100E mutations decrease the stability of IHH-N [15] and verified that these two mutations affect calcium binding because they are close to the calcium-binding groove and are involved in the interaction with the receptor and binding partners [28]. Therefore, A198, K199, T200, and even K191, which is also close to the zinc/calcium-binding groove, may affect protein stability by directly or indirectly binding with zinc or calcium or both. However, this hypothesis requires further in-depth research.

The HH family consists of secretory signal proteins, whose long-range signalling is mainly controlled by the formation of soluble multimers. Therefore, multimerization is crucial for the normal biological function of HH proteins. In view of the *C*-terminal binding model, S195, A196, and A197 promote multimerization by binding to another IHH-N molecule at the zinc-binding site. This study proved that SAA was pivotal for multimer formation and signal activity by using gel-filtration chromatography and cell-based AKP assay.

Cholesterol and palmitic acid modifications were identified to be essential for multimer formation. The absence of palmitic acid or cholesterol modification prevents the multimer formation of HH-N proteins [9,29]. In our study, gel-filtration chromatography results showed that a small amount of multimers could still be detected in tubes 13–15 in SAA truncation, indicating that SAA truncation has normal lipid modification. Cell immunofluorescence results also confirmed this point as ΔSAA could be anchored on the cell membrane, similar to WT, as shown in Figure S6 [30,31]. These results implied that the multimer decrease in ΔSAA was not related to lipid deficiency.

Based on the 3D structure, the essential function of the SHH-N *N*-terminus in multimerization [13] has given new indication that not only lipid modifications but also the interaction between two symmetry-related IHH-N affect multimer formation. Our data show that the highly conserved *C*-terminus of IHH-N was also crucial for multimer formation, thus providing a new standpoint for the study of multimerization. Furthermore, given the published SHH crystal structure [16], A194 and K195 might be involved in SHH multimerization as two key amino acids for the interaction between two symmetry-related SHH-N molecules.

IHH is a secreted protein of the HH family that mainly plays an important regulatory role in cartilage formation and development. IHH is highly conserved in different species, especially the *N*-terminal signal fragment, which is 98% conserved in mice and humans. The highly conserved *C*-terminus of IHH-N, which is located near the cleavage and cholesterol-modifying sites, has sparked the research interest of the authors. This study clearly described the important role of the *C*-terminus in self-cleavage, protein stability, and multimer formation in accordance with the crystal structure of the IHH-N protein, thereby providing an important basis for further elaboration of the mechanism of self-cleavage and multimer formation.

## Data Availability

All data are contained within the manuscript.

## Supplementary Materials

The following are available online at https://www.mdpi.com/article/10.3390/biom11060792/s1, Figure S1: Conservation of the *C*-terminal region of HH-N, Figure S2: *N*-terminal binding model between two molecules of IHH-N, Figure S3: Structural simulation of SAA truncation and AKT truncation proteins, Figure S4: Analysis of EGFP-IHH fusion proteins’ auto-processing, Figure S5: Acrocapitofemoral dysplasia-related mutant V190A affects protein self-cleavage and stability, Figure S6: Cell immunofluorescence of SAA truncation, Table S1: Primers used in this study.
