# Supplementary material for: Highly Conserved C-Terminal Region of Indian Hedgehog N-Fragment Contributes to Its Auto-Processing and Multimer Formation
Source: Biomolecules. 2021 May 25;11(6):792. doi: 10.3390/biom11060792 (PMC8227148; doi:10.3390/biom11060792)
Supplement: Supplementary file 1 [file biomolecules-11-00792-s001.zip › biomolecules-1195526-supplementary.pdf]

Highly Conserved *C*-Terminal Region of Indian Hedgehog *N*-Fragment Contributes to Its auto-processing and Multimer Formation

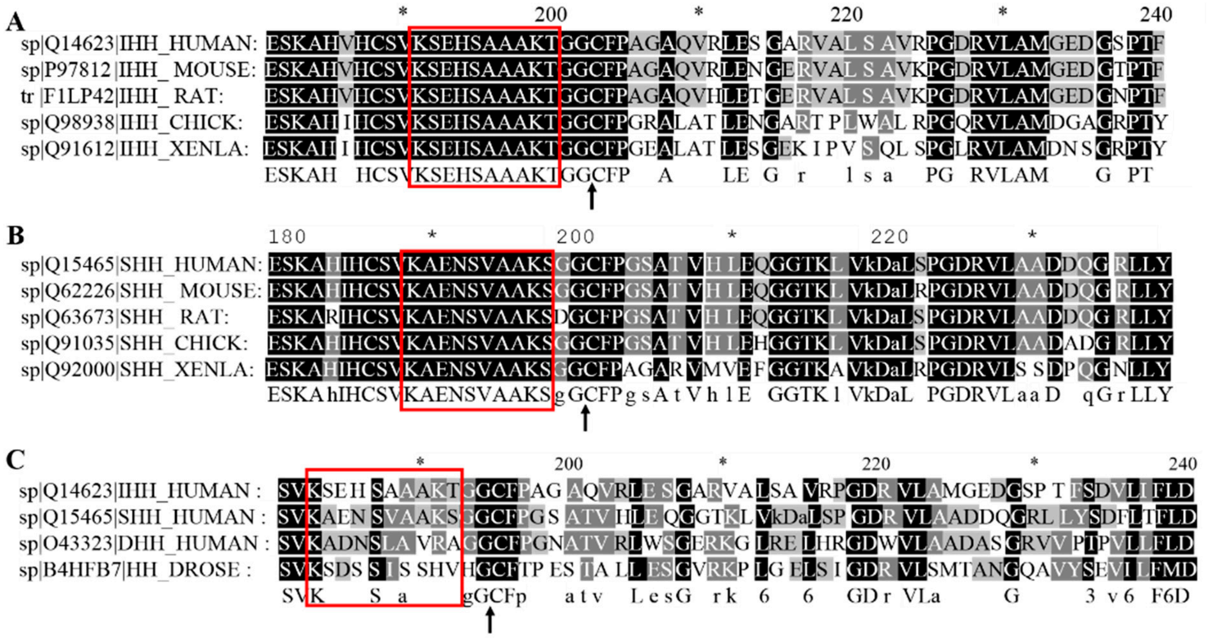

Figure S1. Conservation of the *C*-terminal region of HH-N. The amino acid sequences of IHH-N (**A**) and SHH-N (**B**) were aligned to highlight their conservation within a *C*-terminal region in five different vertebrates, including *Homo sapiens*, *Mus musculus*, *Rattus norvegicus*, *Gallus gallus*, and *Xenopus laeviswere*. (**C**) Conservative analysis between human HH family and Drosophila HH protein. The results showed that DHH, SHH and IHH were conservative to some degree, but the HH protein was less conservative. Self-cleavage and cholesterol modification sites were marked by arrows. The 10 amino acids studied in this work were marked with a red circle.

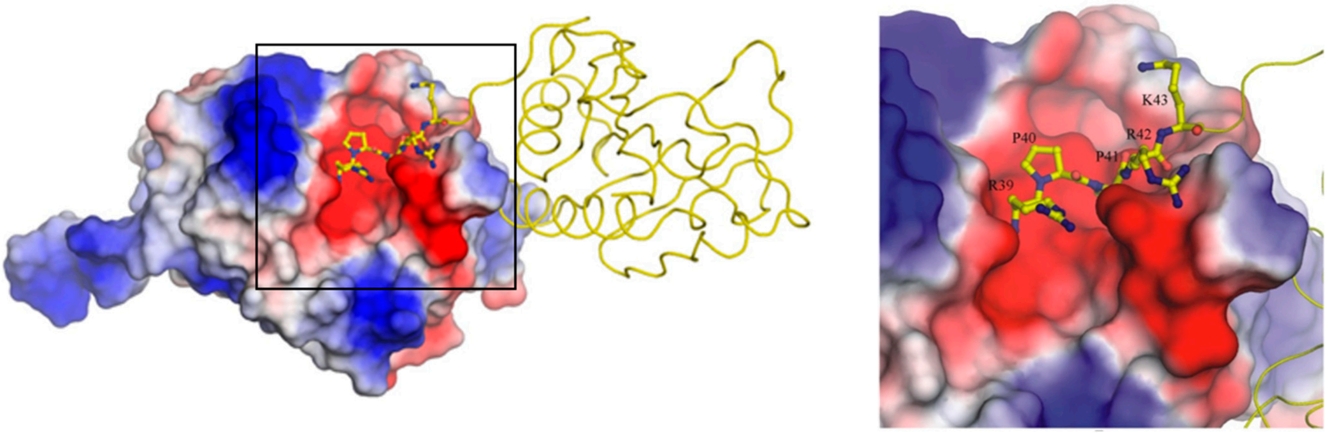

***N*-terminal binding model**

Figure S2. *N*-terminal binding model between two molecules of IHH-N. The *N*-terminal binding model showed that the amino terminus (R39, P40, P41, R42, and K43) of molecule A (pictured as backbone trace) was located at the zinc binding site of molecule B (pictured with the molecular surface area colored by electrostatic potential).

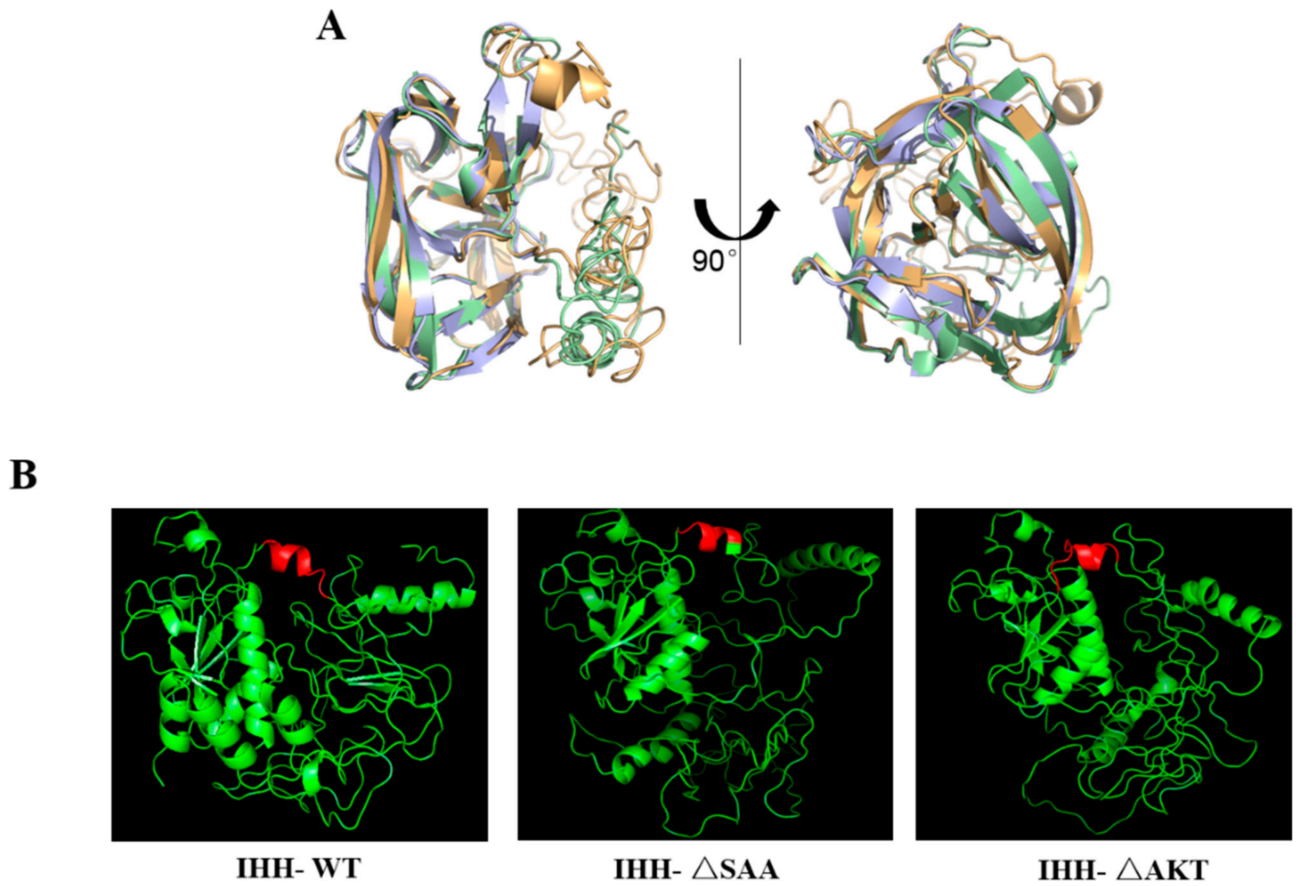

Figure S3. Structure simulation of SAA truncation and AKT truncation proteins. (**A**) The robustness of the predicted human IHH-C (green) and SHH-C (orange) structure with published structure of drosophila HH-C (blue). (**B**) The structure prediction results indicated that truncated SAA or AKT disrupted minimally the overall folding and displayed the rough normally presentation of self-cleavage sites comparing with WT. (the red regions indicate SAAAKTGGC in WT, AKTGGC in SAA truncation, and SAAGGC in AKT truncation, respectively).

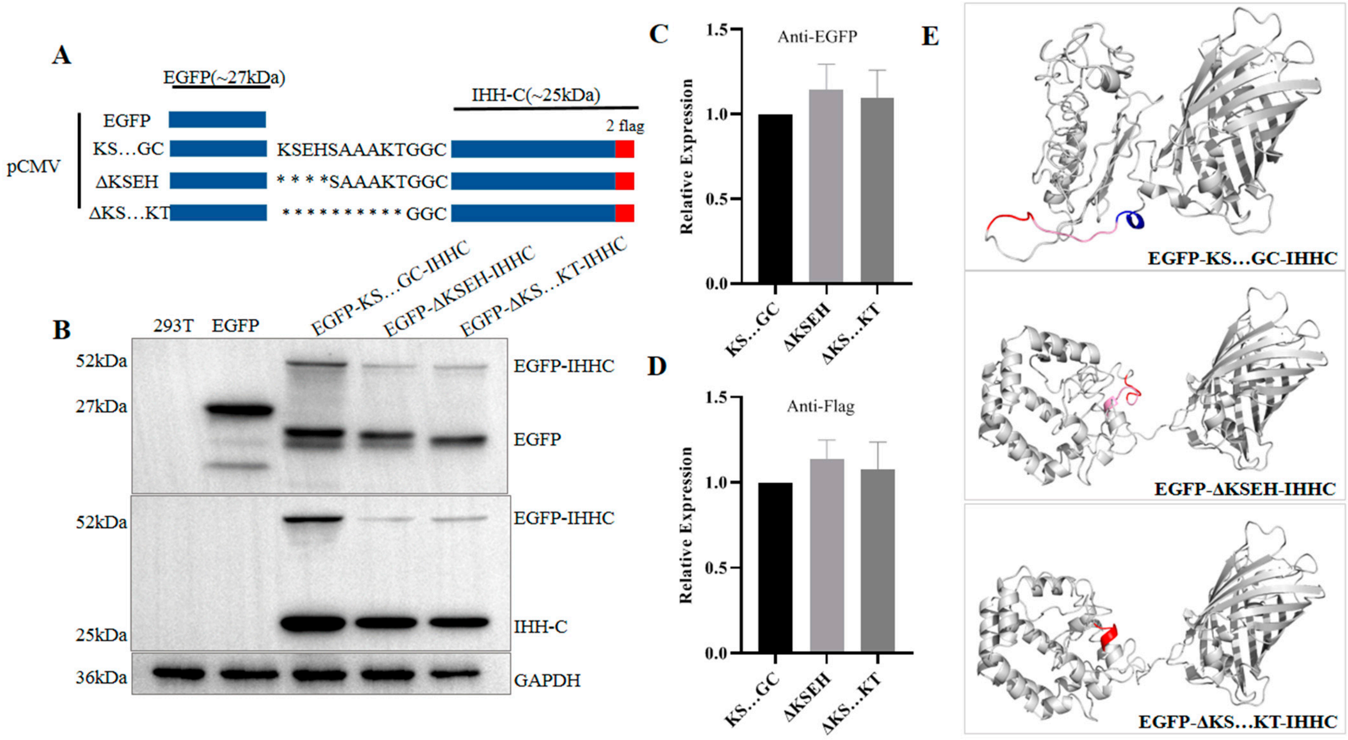

Figure S4. Analysis of EGFP-IHH fusion proteins auto-processing. (**A**) Schematic of three fusion proteins, which replaced IHH-N directly with EGFP but retained the highly conserved *C*-terminus. *truncated amino acids. (**B**) Immunoblots showed the expression of fusion proteins in HEK293 cells. (**C**,**D**) Statistical analysis（n = 3）revealed that K191, S192, E193, and H194 deletion did not affect the self-cleavage of EGFP-IHH precursor, although the 10 conservative amino acids were deleted simultaneously. (**E**) The 3D structure prediction of the fusion protein. The right structure was EGFP, while the left one was IHH-C. Blue, KSEH, Pink, SAAAKT, Red, GGCF. The both IHH-C and EGFP presented relatively independent and intact spatial structures, with the self-cleavage site exposing on the surface all the time.

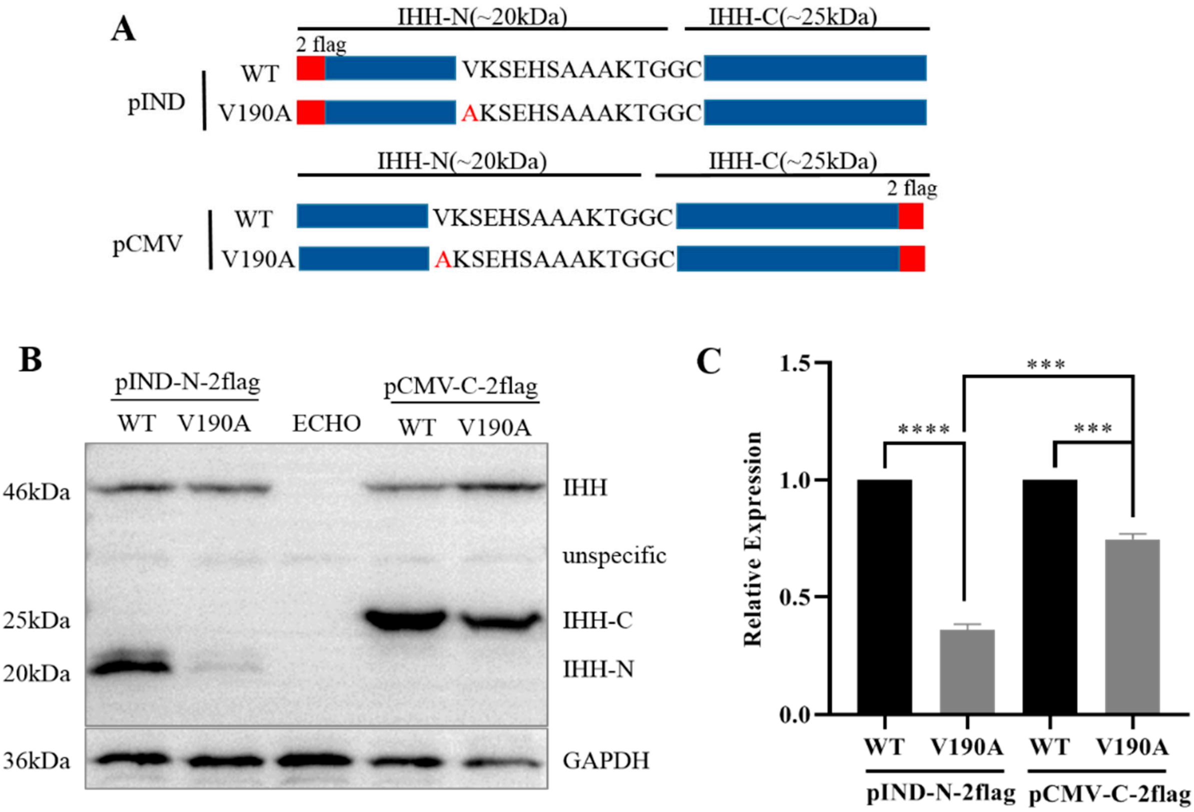

Figure S5. Acrocapitofemoral dysplasia-related mutant V190A affects protein self-cleavage and stability. (**A**) Schematic of V190A mutation. Red letters indicate replaced amino acid. (**B**,**C**) Western blot and quantitative statistical analysis showed that V190A slightly affects self-cleavage but significantly reduces the stability of IHH-N. Data are mean ± s.e.m, $n = 3$, *** $p < 0.001$, **** $p < 0.0001$.

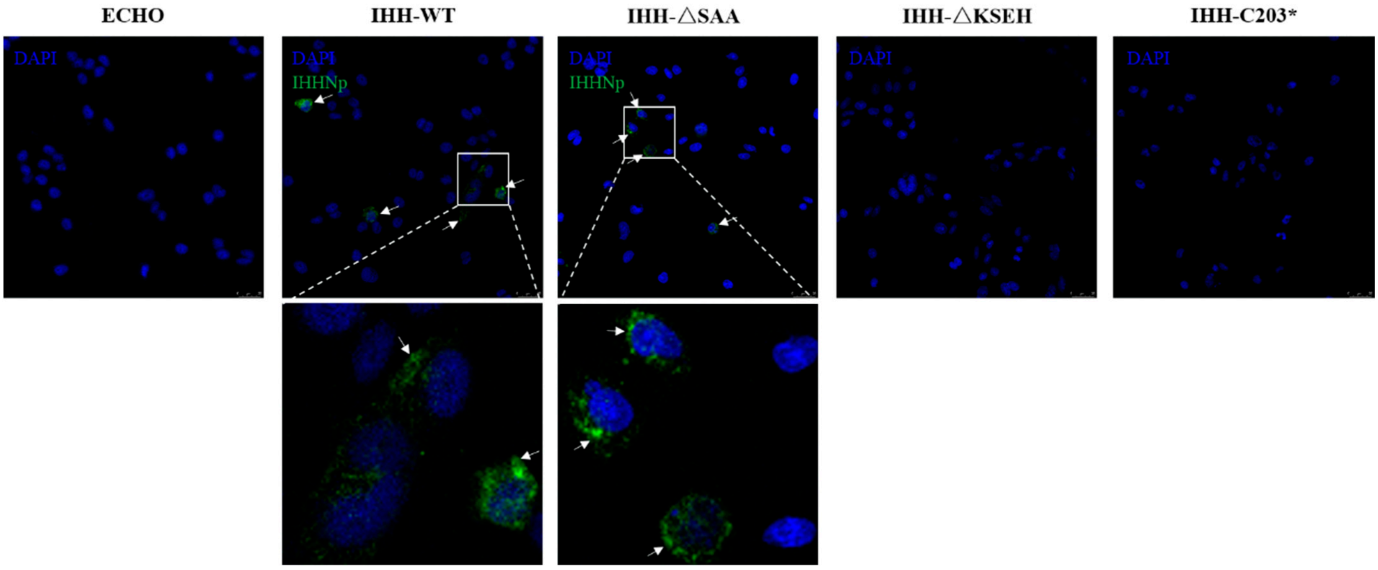

Figure 6. Cell immunofluorescence of SAA truncation. The results showed that IHHNp can be anchored on the cell membrane in SAA truncation, which means it had normal lipid modification as similar as WT. IHH-ΔKSEH without self-cleavage reaction and C203* with cholesterol modification deficiency as negative controls showed no fluorescent signal on the cell membrane. Blue, DAPI, Green, IHHNp. The white arrow, IHHNp on the cell membrane. Scale bars, 25 μm.

Table 1. Primers used in this study.

Primer Name	Primer sequence (5'–3')
pCMV-IHH-C-2flag-F	CCGCTCGAGATGTCTCCCGCCCGGCT
pCMV-IHH-C-2flag-R	GCTCTAGACTACTTATCGTCGTCATCCTTGTAATCCTTATCGT CGTCATCCTTGTAATCGCTCCCTGCCCCGGACAT
IHH-ΔSAA-F	CAAGTCCGAGCACGCCAAGACGGGC
IHH-ΔSAA-R	GCCCGTCTTGGCGTGCTCGGACTTG
IHH-ΔSA…KT-F	GCACGGCGGCTGCTTCCCTG
IHH-ΔSA…KT-R	GCCGCCGTGCTCGGACTTGA
IHH-ΔKS…KT-F	TCCGTCGGCGGCTGCTTCCCTG
IHH-ΔKS…KT-R	CCGCCGACGGAGCAATGCACG
IHH-ΔKSEH-F	CACGTGCATTGCTCCGTCTCGGCCGCAGCCAAGACG
IHH-ΔKSEH-R	CGTCTTGGCTGCGGCCGAGACGGAGCAATGCACGTG
IHH-4A-F	TGCTCCGTCGCGGCCGCGGCCTCGGCCGCA
IHH-4A-R	TGCGGCCGAGGCCGCGGCCGCGACGGAGCA
IHH-K191A-F	GTGCATTGCTCCGTCGCGTCCGAGCACTCGGC
IHH-K191A-R	GCCGAGTGCTCGGACGCGACGGAGCAATGCAC
IHH-S192A-F	TTGCTCCGTCAAGGCCGAGCACTCGGC
IHH-S192A-R	GCCGAGTGCTCGGCCTTGACGGAGCAA
IHH-E193A-F	CCGTCAAGTCCGCGCACTCGGCCGC
IHH-E193A-R	GCGGCCGAGTGCGCGGACTTGACGG
IHH-H194A-F	CGTCAAGTCCGAGGCCTCGGCCGCAGCC
IHH-H194A-R	GGCTGCGGCCGAGGCCTCGGACTTGACG
pCDNA-6xMyc-SHH-F	CGGGATCCGCTGGACTTGACCGCCATG
pCDNA-6xMyc-SHH-R	CCCAAGCTTATGCTGCTGCTGGCGAGATGTC
SHH-ΔKAEN-F	CGATTTGGCCGCCACCGACACCGAGCAGTGGATATG
SHH-ΔKAEN-R	CATATCCACTGCTCGGTGTCGGTGGCGGCCAAATCG
IHH-K199A-F	CGAGCACTCGGCCGCAGCCGCGACGGGCGGCTGCTTCCCTG
IHH-K199A-R	CAGGGAAGCAGCCGCCCGTCGCGGCTGCGGCCGAGTGCTCG
IHH-T200A-F	CGAGCACTCGGCCGCAGCCAAGGCGGGCGGCTGCTTCCCTG
IHH-T200A-R	CAGGGAAGCAGCCGCCCGCCTTGGCTGCGGCCGAGTGCTCG
IHH-ΔAKT-F	CGAGCACTCGGCCGCAGGCGGCTGCTTCCCTG
IHH-ΔAKT-R	CAGGGAAGCAGCCGCCTGCGGCCGAGTGCTCG
EGFP-F	CCGCTCGAGATGGTGAGCAAGGGCGAGG
EGFP-IHH-C-R	GTGGTTTGTCCAAACTCATCAATGTATCTT
EGFP-ΔKSEH-IHHC-F	GAGCTGTACAAGAAGTCCGAGCAC
EGFP-ΔKSEH-IHHC-R	GTGCTCGGACTTCTTGTACAGCTC
EGFP-ΔKS…KT-IHHC-F	AGCTGTACAAGGGCGGCTGCTT
EGFP-ΔKS…KT-IHHC-R	AAGCAGCCGCCCTTGTACAGCT
IHH-V190A-F	GTGCATTGCTCCGCCAAGTCCGAGCACTCGGC
IHH-V190A-R	GCCGAGTGCTCGGACTTGGCGGAGCAATGCAC
IHH-C203*-F	CCAAGACGGGCGGCTGATTCCCTGCCGGAG
IHH-C203*-R	CTCCGGCAGGGAATCAGCCGCCCGTCTTGG
pIND-seq-F	TCTGAATACTTTCAACAAGTTACCG
pIND-seq-R	GGGAGGGGCAAACAACAGATGG
pCMV-seq-F	TCTCTCCACAGGTGTCCACTCC
pCMV-seq-R	CAAACTCATCAATGTATCTTAT
SHH-seq-F	CTGGAGCAGGGCGGCACCAAGC
